# JCPyV T-Antigen Activation of the Anti-Apoptotic Survivin Promoter—Its Role in the Development of Progressive Multifocal Leukoencephalopathy

**DOI:** 10.3390/v12111253

**Published:** 2020-11-03

**Authors:** Luis Del Valle, Thersa Sweet, Amanda Parker-Struckhoff, Georgina Perez-Liz, Sergio Piña-Oviedo

**Affiliations:** 1 Neurological Cancer Research, Stanley S. Scott Cancer Center, Departments of Medicine & Pathology, Louisiana State University Health, New Orleans, LA 70112, USA; 2 Infectious Diseases & AIDS Epidemiology, Department of Epidemiology & Biostatistics, Drexel University Dornsife School of Public Health, Philadelphia, PA 19104, USA; ts36@drexel.edu; 3 Neurological Cancer Research, Stanley S. Scott Cancer Center, Louisiana State University Health, New Orleans, LA 70112, USA; astruckh@amgen.com; 4 A.J. Drexel Autism Institute, Drexel University, Philadelphia, PA 19104, USA; gmp69@drexel.edu; 5 Department of Pathology, College of Medicine, University of Arkansas for Medical Sciences, Little Rock, AR 72205, USA; spinaoviedo@uams.edu

**Keywords:** JC polyomavirus, survivin, progressive multifocal leukoencephalopathy, ChIP assay, proximity ligation assay, JCPyV, T-antigen

## Abstract

Progressive Multifocal Leukoencephalopathy (PML) is a fatal demyelinating disease of the CNS, resulting from the lytic infection of oligodendrocytes by the human neurotropic polyomavirus JC (JCPyV), typically associated with severe immunocompromised states and, in recent years, with the use of immunotherapies. Apoptosis is a homeostatic mechanism to dispose of senescent or damaged cells, including virally infected cells, triggered in the vast majority of viral infections of the brain. Previously, we showed upregulation of the normally dormant anti-apoptotic protein Survivin in cases of PML, which—in vitro—resulted in protection from apoptosis in JCPyV-infected primary cultures of astrocytes and oligodendrocytes. In the present study, we first demonstrate the absence of apoptotic DNA fragmentation and the lack of caspase activity in 16 cases of PML. We also identified the viral protein large T-Antigen as being responsible for the activation of the Survivin promoter. Chromatin Immunoprecipitation assay shows a direct binding between T-Antigen and the Survivin promoter DNA. Finally, we have identified the specific region of T-Antigen, spanning from amino acids 266 and 688, which binds to Survivin and translocates it to the nucleus, providing evidence of a mechanism that results in the efficient replication of JCPyV and a potential target for novel therapies.

## 1. Introduction

The human neurotropic virus JC (JCPyV), a member of the Polyomaviridiae family of DNA viruses, which also includes BKPyV, SV40 and the Merkel cell polyomavirus (MCPyV) [1], is the well-established opportunistic etiological agent of the fatal, demyelinating disease of the brain Progressive Multifocal Leukoencephalopathy (PML). Once considered a rare entity, PML became a common neurologic complication in Acquired Immunodeficiency Syndrome (AIDS) patients after the Human Immunodeficiency Virus-1 (HIV-1) pandemic in the 1980s and is now considered an AIDS-defining condition [2,3]. Since then, the incidence of PML has significantly decreased thanks to the effective control of HIV-1 infections with antiretroviral therapy [4]; however, a new peak of cases has emerged with the use of immunotherapies for auto-immune conditions such as multiple sclerosis and Chron’s disease [5,6]. The pathological features of PML include the presence of demyelinating foci located in the gray–white matter junction of the brain, predominantly in frontal and parieto-occipital lobes. Histologically, PML consists of areas of severe demyelination with multiple foamy macrophages, microglial nodules, perivascular cuffing of lymphocytes, both findings of any CNS viral infection, and the pathognomonic cells of the disease: enlarged oligodendrocytes harboring intra-nuclear inclusion eosinophilic bodies and bizarre atypical astrocytes, reminiscent of malignant glial cells [7]. JCPyV is a small (38–40 nm), non-enveloped virus with a double-stranded DNA and a circular genome of 5130 nucleotides that can be divided into three regions: a non-coding regulatory region that controls the initiation of replication and viral transcription, which is located between an early and a late transcriptional region. The early region contains the codifying sequences for the large and small T-Antigens (T-Ag and t-Ag), and the late region encodes for the late accessory Agnoprotein, and the capsid proteins (VP-1, VP-2 and VP-3 [8]).

Apoptosis, or programmed cell death, is a mechanism necessary to eliminate senescent cells in order to keep a balance of tissue renewal. It is also a crucial mechanism for disposing of damaged and potentially harmful cells, including virus-infected cells, in an effort to prevent the propagation and spreading of viral infections. On the other hand, viral pathogens can induce apoptosis, necrosis, or both types of cell death on infected target cells after completing replication and virion formation. Several viruses can gain access to the Central Nervous System (CNS), produce infection and trigger programmed cell death [9]. Accordingly, the presence of apoptosis has been described in viral encephalitis due to herpes simplex [10] and cytomegalovirus [11], measles [12,13], poliovirus [14], rabies [15], human immunodeficiency virus-1 (HIV-1) [16,17,18], Coxsackie viruses A and B [19,20], and arboviruses [21]. However, apoptosis has not been convincingly demonstrated in cases of Progressive Multifocal Leukoencephalopathy in humans.

Although the lytic death of infected oligodendrocytes is the cause of demyelination in PML, it is noteworthy that apoptosis has not been described morphologically or observed utilizing ultrastructural studies in JCPyV-infected cells [22]. In fact, in comparison to other CNS viral infections in which apoptosis seems to be a common occurrence, JCPyV has been shown to induce non-apoptotic cell death in in vitro experiments [23]. In a previous study, our group has demonstrated that JCPyV-infection induces the expression of Survivin, a member of the inhibitor of apoptosis (IAP) proteins, at the mRNA and protein levels [24]. Survivin is a 142-amino acid, 16.5-kD protein that is widely expressed during embryogenesis but becomes silenced in adult or fully differentiated tissues [25]. Its functions include the inhibition of extrinsic and intrinsic apoptotic pathways, and a role in mitotic spindle formation and cell division [26,27,28]. Expression of the protein is finely regulated through the cell cycle, showing a peak at the G2/M phase and low levels at G1 [27]. Activation or repression of Survivin is governed by its promoter, which contains three cycle-dependent elements (CDE), one cell cycle homology region (CHR), a 5-C-phospahte-G-3’ (CpG) island, and several binding sites for the Sp1 transcription factor [29]. Interestingly, Survivin promoter activation can be induced by several cell cycle- and non-cell cycle-related proteins [30,31,32,33,34,35,36]. Similarly, some transcription factors may activate the Survivin promoter and favor aberrant, cell cycle-independent up-regulation of the protein and prevent apoptotic cell death [37]. This and other factors may promote cancer development since Survivin overexpression has been observed in several malignancies [38,39].

In our previous study, we showed the presence of Survivin in the nuclei of JCPyV-infected oligodendrocytes and in the cytoplasm of JCPyV-infected astrocytes, while both phenotypes of glial cells outside plaques of demyelination remained negative. Then, we corroborated the activation of transcription and Survivin production in primary cultures of astrocytes and oligodendrocytes upon JCPyV infection. The activation of the normally dormant Survivin resulted in the inhibition of natural and staurosporine-induced apoptosis and, finally, to prove that the protection from apoptosis upon JCPyV infection was Survivin related, siRNA inhibition of the anti-apoptotic protein resulted in catapulting the number of apoptotic cells [24]. Based on these results, we hypothesized that the activation of Survivin may be beneficial for JCPyV in order to increase cell survival and thus allow time for an efficient viral replication and propagation of viral particles once the lytic cycle is completed.

Indeed, viruses have developed ways to prevent the programmed cell death of infected cells in order to sustain cell viability until completion of their infectious cycles. This virus-mediated resistance may be coordinated by viral and/or cellular mediators through direct activation or overexpression of anti-apoptotic proteins, including Survivin [40,41,42,43,44]. In fact, Survivin up-regulation has also been observed in viral infection processes, including PML, as mentioned above [24,45]. However, the mechanism by which Survivin production is activated in PML remains elusive, but is logical to assume it depends upon expression of JCPyV regulatory proteins, such as large T-Antigen or the late accessory Agnoprotein. Within this context, it has been previously shown that T-Antigen can induce the activation or repression of several cellular genes by direct or indirect interaction with enhancer/promoter regions, including in its own promoter [46,47], suggesting that the viral early protein may be responsible for the Survivin over-expression found in PML. In this manuscript, we explore the molecular mechanisms involved in the activation of Survivin by JCPyV proteins.

## 2. Materials and Methods

Histology and immunohistochemistry: A total of sixteen formalin-fixed, paraffin-embedded PML samples, HIV-E and control brains have been collected from the pathology departments of the University of Laussane, Switzerland, Thomas Jefferson University (Philadelphia, PA, USA), Temple University (Philadelphia, PA, USA), Louisiana State University (New Orleans, LA, USA), and the Manhattan Brain Bank at Mount Sinai Medical center (New York, NY, USA). Hematoxylin & Eosin staining was performed for routine histological evaluation of sixteen PML samples. Immunohistochemistry was performed using the avidin–biotin peroxidase methodology, according to the manufacturer’s instructions (Vectastain ABC Elite Kit, Vector Laboratories, Burlingame, CA, USA). Our modified protocol includes deparaffination in xylenes, rehydration through descending grades of ethanol up to water, non-enzymatic antigen retrieval with 0.01 M sodium citrate buffer pH 6.0 at 95 °C for 25 min, endogenous peroxidase quenching with 3% H_2_O_2_ in methanol, blocking with normal horse serum (for mouse monoclonal antibodies) or normal goat serum (for rabbit polyclonal antibodies) and incubation with primary antibodies overnight at room temperature in a humidified chamber. After rinsing in Phosphate Buffer Solution (PBS), sections were incubated with biotinylated secondary antibodies for 1 h, followed by incubation with avidin–biotin peroxidase complexes for 1 h. Finally, the peroxidase was developed with diaminobenzidine (Boehringer, Mannheim, Germany) for 3 min, and the sections were counterstained with Hematoxylin and mounted with Permount (Fisher Scientific, Pittsburgh, PA, USA). Antibodies for apoptotic and anti-apoptotic proteins were as follows: a rabbit polyclonal anti-cleaved caspase-3 (Asp175, 1:400 dilution, Cell Signaling Technology, Danvers, MA, USA); a rabbit polyclonal anti-cleaved caspase-9 (Asp330, 1:250, Cell Signaling Technology, Danvers, MA, USA); and a mouse monoclonal anti-Survivin (Clone D-8, 1:100, Santa Cruz Biotechnology, Santa Cruz, CA, USA). Antibodies against JCPyV proteins included a mouse monoclonal anti-SV40 T-Antigen (Ab-2) (Clone pAb416, 1:100, Calbiochem, San Diego, CA, USA), which cross-reacts with JCPyV T-Antigen, and a rabbit polyclonal antibody against the capsid protein VP-1 (CR7947, 1:4000, courtesy of Dr. Walter Atwood, Brown University, Providence RI). Photomicrographs were taken with an Olympus DP72 Digital Camera using an Olympus BX70 microscope (Olympus, Center Valley, PA, USA).

TUNEL assay: A peroxidase in situ Terminal dUTP Nick-End Labeling (TUNEL) assay was performed in archival paraffin-embedded PML samples. Tissues were rehydrated as previously described for immunohistochemistry and the TUNEL assay was performed according to the manufacturer’s instructions (ApopTag^®^ Peroxidase In Situ Apoptosis Detection Kit, Chemicon International, Inc., Billerica, MA, USA). Slides were developed with diaminobenzidine (Boehringer), counterstained with HHematoxylin and mounted with Permount (Fisher Scientific, Pittsburgh, PA, USA).

Infection of primary astrocytic cultures: Human astrocytes and growth medium were purchased from Cambrex Bio Science, Inc. (Clonetics astrocyte cell systems, Walkersville, MD, USA). Cells were infected with 100 hemagglutination units of the Mad1/SVEΔ strain of JCPyV, equivalent to a multiplicity of infection (m.o.i.) of 1, in the absence of serum for 3 h at 37 °C. The features of this hybrid JCPyV have been previously described [24]. Cells were washed and re-fed with growth media supplemented with 15% Fetal Bovine Serum (FBS) after infection. Efficiency of viral gene expression and viral replication was evaluated by Western blot and immunocytochemistry using antibodies against T-Antigen and VP-1, respectively.

Transfection of mice and human tumor cell lines: 100-mm dishes with 1 × 10^6^ BsB8 cells were transiently transfected with 10 μg of a human Survivin expressing plasmid (pCMV-Survivin), and same quantity of U87-MG cells were transiently co-transfected with same 10 μg of pCMV-Survivin and 10 μg of a expressing vector containing the JCPyV large T-Antigen, pcDNA3.1/Zeo(+)/JCT (KpnI-EcoRI into pcDNA3.1/Zeo(+)) using the CaPO_4_ transfection method [48]. DNA amounts were standardized for each cell line using a pcDNA3.1 empty vector.

Chromatin Immunoprecipitation assay: A Chromatin Immunoprecipitation (ChIP) assay was performed according to the manufacturer’s instructions (EZ-ChIP, Millipore/Sigma, St. Louis, MO, USA). Primary cell lines were cultured as described above. After fixation, 1 × 10^6^ cells were washed with PBS. Chromatin was isolated by the addition of lysis buffer (1% SDS, 10 mM EDTA, 50 mM Tris-HCl, pH 8.1) with 1 mM phenylmethylsulfonyl fluoride, 1 mg/mL aprotinin, and 1 mg/mL pepstatin A, followed by disruption with a Dounce homogenizer. Lysates were sonicated and the DNA sheared to an average length of ~350–500 bp. Genomic DNA (input) was prepared by treating aliquots of chromatin with RNase, proteinase K and heat for de-crosslinking, followed by ethanol precipitation. Pellets were resuspended and the resulting DNA was quantified on a NanoDrop spectrophotometer. Extrapolation to the original chromatin volume allowed quantitation of the total chromatin yield. An aliquot of chromatin (30 μg) was precleared with protein, and the described mouse monoclonal anti-T-Antigen was utilized. Pellets were resuspended in TE buffer and subjected to PCR amplification using forward and reverse primers (5′-GATTACAGGCGTGAGCCACT-3′ and 5′-ATCTGGCGGTTAATGGCGCG-3′) selected from the survivin core promoter sequence (−253 to −234 and −11 to +10, respectively).

Survivin promoter constructs and Luciferase assay: In order to identify the region of the Survivin promoter responsible for the activation of the Survivin gene during the course of JCPyV infection, we cloned different deletion mutants of the promoter into a pGL3 vector to be used for transcription studies. Primary human astrocytes were then transiently transfected with these constructs in the presence and absence of plasmids expressing T-Antigen and Agnoprotein. All cells were also transfected with pRL-TK-LUC and were used to normalize the Luciferase data. Promoter activity was analyzed by Luciferase assay, using the Promega kit as recommended by the manufacturers. The relative light units (RLU) obtained from Luciferase emission were divided by the RLU from Renilla emission to obtain the normalized data points. All transfection and Luciferase assays were performed three times in order to obtain statistically significant data.

Primary fetal astrocytes were plated in 100-mm dishes at 80% confluence and maintained in media. Cells were transfected with 10 μg of firefly luciferase reporter construct and 10 μg of *Renilla* Luciferase construct (pRL-TK, Promega, Madison, WI, USA) along with increasing amounts of JCPyV T-Antigen expression plasmid. Cells were harvested 36 h later, lysed, and dual Luciferase assays were performed following the manufacturer’s instructions. Luciferase activity was normalized to total protein levels, as well as to *Renilla* Luciferase activity.

Western blot and co-immunoprecipitation: For these experiments, we used the commercially available malignant glial cell line U87-MG, and BsB8 cells, a clonal cell line isolated from JCPyV transgenic mice brain tumors [49]. Transiently transfected U87-MG and BsB8 cells were lysed in TNN buffer (50 mM Tris, pH 7.4, 150 mM NaCl, 5 mM MgCl_2_, 0.5% NP-40) and centrifuged for 10 min at 4 °C. Whole cell extracts (150 μg) were immunoprecipitated overnight at 4 °C with a mouse monoclonal anti-Survivin antibody (10 μL, Santa Cruz Biotechnology, Santa Cruz, CA, USA) using 50 μL of protein A-Sepharose beads (GE Healthcare, Buckinghamshire, UK). The following day, extracts were separated using a 10% SDS-denaturing polyacrylamide gel and transferred to a pure nitrocellulose membrane (Trans blot^®^ Transfer Medium, Bio-Rad Laboratories Inc., Hercules, CA, USA). A monoclonal mouse antibody against SV40 T-Antigen (Calbiochem) was incubated overnight at 4 °C at a 1:1000 dilution, and an anti-mouse horseradish peroxidase-conjugated secondary antibody (Thermo Scientific, Waltham, MA, USA) was used at a 1:10,000 dilution. Negative controls were carried out by incubating same amounts of protein extracts with 10 μL of a non-specific mouse IgG and/or in absence of primary antibody. All experiments were performed in duplicate.

Double-labeling immunofluorescence: Deparaffination and rehydration of tissues were performed as described for immunohistochemistry above. After overnight incubation with the first primary antibody (a rabbit monoclonal anti-Survivin, abcam, cloneEP2880Y, 1:500 dilution), sections were rinsed with PBS, and an Alexa Fluor 488-tagged anti-rabbit secondary antibody was incubated for 2 h at room temperature in the dark. After washing thoroughly with PBS, a second primary antibody (mouse monoclonal anti-SV40 T-Antigen) was incubated overnight at RT. Finally, a second Alexa Fluor 568-conjugated anti-mouse secondary antibody was incubated for 2 h at RT in the dark, and sections were coverslipped with an aqueous-based mounting media (Vectashield^®^ Hard Set with DAPI; Vector Laboratories). Visualization and image capture was done on an Olympus FV1000 confocal microscope and Fluoview software.

Proximity Ligation Assay: An in situ Proximity Ligation Assay was performed according to the manufacturer’s instructions (DuoLink, Millipore-Sigma, Burlington, MA, USA). Briefly, primary glial cell cultures, infected with JCPyV as described above, were fixed and incubated with a primary anti-mouse anti-T-Antigen antibody and after through rinsing with a rabbit monoclonal anti-Survivin recombinant antibody. Next, oligonucleotide-labeled secondary anti-mouse and anti-rabbit antibodies were incubated. Next, hybridizing connector probes are incubated with a ligase in a ligation buffer. Amplification is done with Polymerase in an amplification buffer. Finally, a DouLink red detection reagent is incubated. Slides were then washed, and coverslipped with an aqueous mounting media containing DAPI. A DouLink PLA Control kit/slide containing SK-OV3 human ovarian cancer cells and antibodies for EGFR-HER2 was used as a positive and negative control (data not shown).

Glutathione S-transferase (GST) pulldown assay using ^35^[S]-radiolabeled in vitro Survivin: GST and GST-T-Antigen fusion proteins (full length and deletion mutants) were obtained from bacterial cultures after sonication (constant duty cycle for 4–5 s, 3 times) in cold NETN buffer (100 mM NaCl, 1 mM EDTA, 20 mM Tris, pH 8.0, 0.5% NP-40) with protease inhibitor cocktail (1:10 dilution, SIGMA-Aldrich, Saint Louis, MO, USA) and lysozyme (1:100 dilution, 200 mg/mL stock). After centrifugation for 30 min at 4 °C, supernatants were incubated with 300 μL of pre-washed glutathione sepharose 4B beads (GE Healthcare) overnight at 4 °C. The next day, GST beads and GST fusion proteins were washed in NETN buffer and eluted in GST elution buffer (50 mM Tris, pH 8.0, 5 mM DTT, 2 mM reduced glutathione) and stored at −70 °C. For the generation of ^35^[S]-radiolabeled in vitro-translated (IVT) Survivin, we used TNT^®^ Quick Coupled Transcription/Translation Systems according to the manufacturer’s instructions (Promega) Survivin mRNA. In total, 5 μL of the ^35^[S]-radiolabeled IVT-Survivin were incubated for 2 h at 4 °C with equal amounts of GST and GST-T-Antigen fusion proteins along with 25 μL of BSA, 25 μL of GST beads, 300 μL of lysis buffer 150 (LB150; 50 mM Tris-HCl, pH 7.4, 150 mM NaCl, 5 mM EDTA, 0.1% NP-40, 50 mM NaF) and mammalian protease inhibitor cocktail (1:100, SIGMA). After this, beads were centrifuged and washed with 300 μL of LB150 with protease inhibitors. GST fusion proteins were then separated in a 10% SDS- denaturing polyacrylamide gel, which was stained with Coomassie Blue stain and rinsed with distilled water. The gel was soaked in Autofluor^®^ (National Diagnostics, Atlanta, GA, USA) for 1 h, dried for another hour, and placed into an X-ray cassette with overnight film exposure at −70 °C. GST was run as a negative control, whereas 1/10 of the ^35^[S]-IVT protein (input) was run as a positive control.

## 3. Results

### 3.1. Expression of Survivin But Not Apoptotic Proteins in JCPyV-Infected Cells of PML

**Figure 1:**
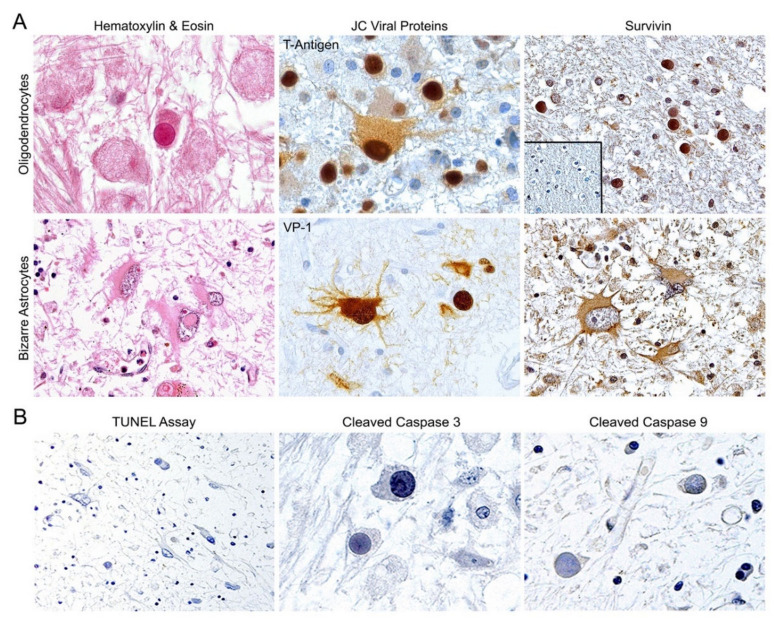
Expression of Survivin and absence of caspase activity in cases of Progressive Multifocal Leukoencephalopathy (PML). (Panel **A**) Pathognomonic histological findings in PML include the presence of enlarged oligodendrocytes harboring intra-nuclear eosinophilic inclusions bodies (upper left panel, H&E, 1000× magnification) and bizarre-shaped, transformed astrocytes (lower left panel, H&E, 400× magnification) within demyelinated plaques. Expression of T-Antigen and the capsid protein VP-1 are observed in infected oligodendrocytes and astrocytes of PML lesions (middle panels, 600×). Immunohistochemical analysis of Survivin demonstrates the anti-apoptotic protein is abundantly expressed in both subtypes of JCPyV-infected cells, oligodendrocytes (upper panel) and astrocytes (lower panel). Importantly, no expression of Survivin was detected in normal brain, nor brains undergoing other pathologies (inset), nor in the astrocytes and oligodendrocytes in non-demyelinated plaques. Survivin is present in the nuclear inclusion bodies and cytoplasm of infected oligodendrocytes, and mostly in the cytoplasm of bizarre astrocytes (right panel, 400×). (Panel **B**) In addition to the up-regulation of Survivin, no detection of DNA laddering (TUNEL assay) was found in any cell type of the disease (right panel, 200×), and the cleaved forms of caspase-3 or caspase-9 were absent in inclusion bodies of infected oligodendrocytes (middle and right panel, 1000×).

Characterization of PML was established by histopathological analysis of Hematoxylin & Eosin (H&E) tissue sections, where frequent hallmark cells of the disease, oligodendrocytes with intranuclear eosinophilic inclusion bodies and bizarre astrocytes, were observed (Figure 1, Panel A, left). Corroboration of the infection of these cells was established by detection of the viral proteins, T-Antigen and capsid VP-1, by immunohistochemistry (Panel A, middle). Expression of T-Antigen is frequently observed in early lesions of PML in the nucleus of oligodendrocytes and in the cytoplasm and nucleus of bizarre astrocytes; conversely, in late lesions, T-Antigen is less frequently observed, but the detection of capsid proteins with an anti-VP1 antibody is a reliable indicator of active JCPyV infection. We performed immunohistochemistry for the anti-apoptotic protein Survivin in 16 cases of PML and in normal human brain tissues. As previously demonstrated by our group [24], robust expression of the anti-apoptotic protein is detected in the intranuclear inclusion bodies and cytoplasm of infected oligodendrocytes, and predominantly in the cytoplasm of bizarre astrocytes (Panel A, right). In contrast, Survivin is absent in adjacent non-affected areas of PML and in adult normal brain samples (Panel A, inset). Furthermore, we performed TUNEL assay for the detection of apoptosis in PML but no DNA laddering was observed in any cells. To corroborate the effects of the presence of Survivin, we performed a TUNEL assay for the detection of apoptosis in our 16 PML samples, and no DNA laddering was observed in any cells in any case. In contrast, apoptosis was detected in two cases of HIV encephalopathy we used as controls. Finally, expression of cleaved caspase-3 and -9 was not detected by immunohistochemistry in JCPyV-infected oligodendrocytes, nor bizarre astrocytes (Panel B). Table 1 shows the results the clinical information of the cases studied and the results from the immunohistochemistry and TUNEL assay.

### 3.2. Activation of the Survivin Promoter by JCPyV T-Antigen

**Figure 2:**
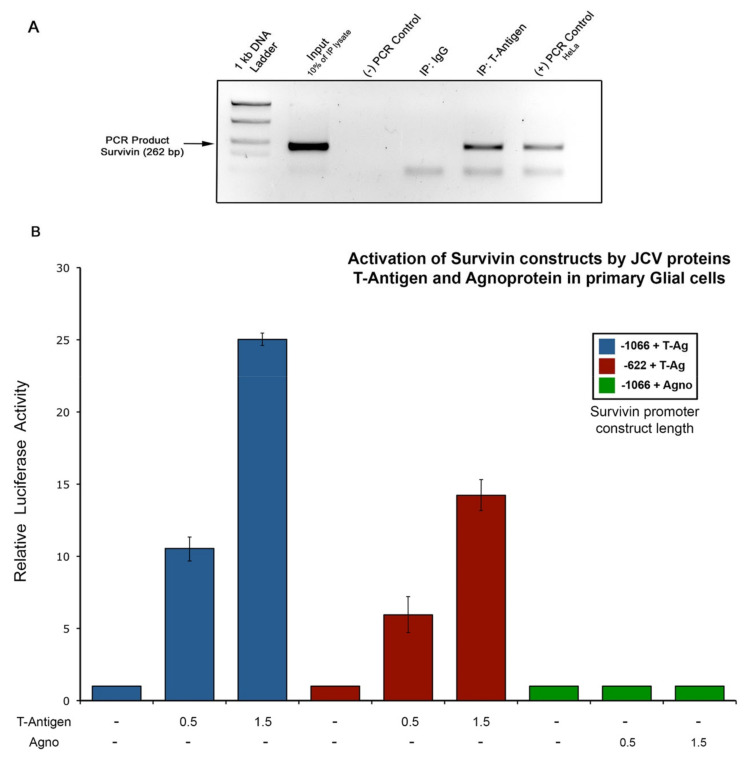
(**A**)Human neurotropic polyomavirus JC (JCPyV) T-Antigen transactivates the Survivin promoter. Chromatin Immunoprecipitation (ChIP assay) of primary astrocytic cell cultures infected with JCPyV shows a direct binding between T-Antigen and the Survivin promoter DNA, demonstrating a physical binding between the two elements. (**B**) Luciferase activity of primary astrocytic cell cultures co-transfected with two Survivin promoter constructs of different lengths (−1066 to +45 and −622 to +45), and either a plasmid expressing T-Antigen or the Agnoprotein, demonstrate a significant transactivation of the Survivin promoter by T-Antigen (Lanes 2, 3 and 5, 6), in comparison to basal Luciferase activity (Lanes 1 and 4). Interestingly, activation of the Survivin promoter region was directly proportional to the concentration amount of T-Antigen (0.5 or 1.5 µg) used with both constructs, independently of their length. In contrast, no changes on Survivin promoter activation occurred when Agnoprotein was present at any concentration (Lanes 8 and 9). These data indicate that up-regulation of Survivin is specifically mediated by T-Antigen and not by the late accessory protein. Luciferase activity was measured 36 h after transfection and the results are expressed as the means ±SD from three experiments.

In order to demonstrate if the early transcriptional protein of JCPyV, T-Antigen, is responsible for the activation of the normally dormant anti-apoptotic Survivin promoter, we performed a Chromatin Immunoprecipitation Assay in primary human glial cells infected with the Mad1/SVEΔ strain of JCPyV. Figure 2A shows the results of the ChIP assay demonstrating a clear band of T-Antigen bound to the Survivin DNA. HeLa cells were used as a PCR positive control. These results constitute the first evidence of the physical binding between T-Antigen and the Survivin promoter. Then, in order to determine if JCPyV T-Antigen or the Agnoprotein are responsible for the activation of Survivin expression in PML, we performed Luciferase assays in the same human fetal glial cell cultures. Primary astrocytes were co-transfected with either the −1066 to +45 (1111 bp length) or the −622 to +45 kb (667 bp length) Survivin promoter constructs driving the Luciferase reporter gene, and with two different amounts (0.5 and 1.5 μg, respectively) of a plasmid expressing either the JCPyV T-Antigen or the Agnoprotein, and Luciferase activity measured after 36 h. Surprisingly, the ectopic production of T-Antigen resulted in significant activation of Survivin promoter transcription compared to basal Luciferase activity (Figure 2B, Lanes 1 to 6). Furthermore, it appears that the activation of the promoter is directly proportional to the amount of T-Antigen present in the cells, as cells transfected with 0.5 μg of LT showed an average of 10-fold activation of the promoter (Lanes 2 and 5), whereas cells transfected with 1.5 μg of LT exhibited an average of 20-fold activation (Figure 2B, Lanes 3 and 6). Another significant observation is the fact that cells transfected with the longer length promoter construct (−1066 to +45, blue bars) were activated more efficiently by T-Antigen compared to the shorter length construct (−622 to +45, red bars). On the contrary, expression of two different amounts of AGNO failed to induce the Luciferase activity of any of the promoter constructs (Lanes 7 to 9). These data strongly suggest that activation of the Survivin promoter and Survivin protein expression most likely occurs due to a T-Antigen-specific mechanism upon JCPyV infection, and also that T-Antigen is sufficient to up-regulate the Survivin promoter region, even in the absence of the complete JCPyV genome.

### 3.3. Co-Localization and Physical Interaction between T-Antigen and Survivin in Cell Cultures

**Figure 3:**
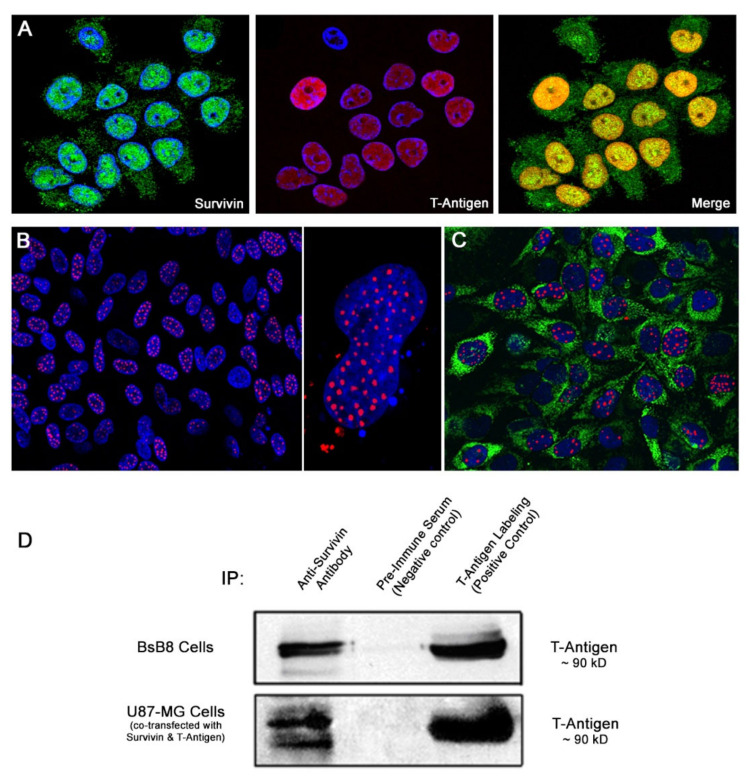
Physical interaction between JCPyV T-Antigen and Survivin in in vitro experiments. (**A**) Double-labeling immunocytochemistry of JCPyV-infected primary astrocytes demonstrates the co-localization of the anti-apoptotic protein Survivin (green) and large T-Antigen (red) in the nuclei of infected cells (All panels original magnification is 200×). (**B**) Proximity Ligation Assay (DuoLink) demonstrates the physical interaction between these two proteins in the nuclei of JCPyV-infected cells (left panel original magnification is 100×, right panel 1200×). (**C**) Furthermore, and to corroborate the phenotype of the infected cells, immunolabeling with Glial Fibrillary Acidic Protein (GFAP) was performed following DuoLink, demonstrating the glial nature of the cells in which the Survivin-T-Antigen interaction occurs. (**D**) Co-immunoprecipitation analyses demonstrate a physical interaction between T-Antigen and Survivin in cell cultures. A band the same size as T-Antigen (~90 kDa, positive control lane) was observed in the protein extracts immunoprecipitated with an anti-Survivin antibody, but not when pre-immune serum was added to the samples (negative control), in two different cell lines (BsB8 and U87-MG). BsB8 is a murine neuroectodermal tumor cell line that expresses constant levels of T-Antigen, and U87-MG is a glial tumor cell line in which transient co-transfection with T-Antigen and Survivin plasmids was performed.

We have previously shown that Survivin mRNA and protein expression is induced upon JCPyV-infection in primary oligodendroglial and astrocytic cell cultures, and that Survivin is mostly found in the nucleus of these cells [24]. Survivin, however, is a predominantly cytoplasmic protein (it contains a nuclear export signal) with still uncertain functions within the nuclear compartment [38,50,51,52]. Interestingly, double labeling immunofluorescence experiments conducted in our JCPyV-infected astrocytic cultures 5 days post-infection, showed expression of T-Antigen in the nuclei of infected cells, in which Survivin was also present, co-localizing in the nucleus (Figure 3A). In contrast, neither Survivin nor T-Antigen expression was found in uninfected cell cultures (data not shown). These observations suggest a direct interaction between both proteins.

To determine if a real physical interaction between JCPyV T-Antigen and Survivin exists, we performed co-immunoprecipitation assays with whole-cell extracts from BsB8 cells transiently transfected with a Survivin plasmid vector. BsB8 cells are a primitive neuroectodermal neoplastic cell line grown from a tumor developed in a JCPyV transgenic mouse, which contains the early gene of the Archetype strain under the control of its own promoter, and expresses high levels of T-Antigen [49]. By Western blot analysis, a band corresponding to T-Antigen was detected in the immuno-complex pulled down using an anti-Survivin antibody, while there was no band observed in the lane containing extract incubated with pre-immune control serum, indicating a specific interaction between these two proteins (Figure 3B). Likewise, extracts from U87-MG cells, a neoplastic cell line of glial origin, co-transfected with a plasmid vector expressing Survivin and the T-Antigen, respectively, yielded similar results, with T-Antigen detection in complex with Survivin (Figure 3B).

### 3.4. Binding of JCPyV T-Antigen to Survivin

**Figure 4:**
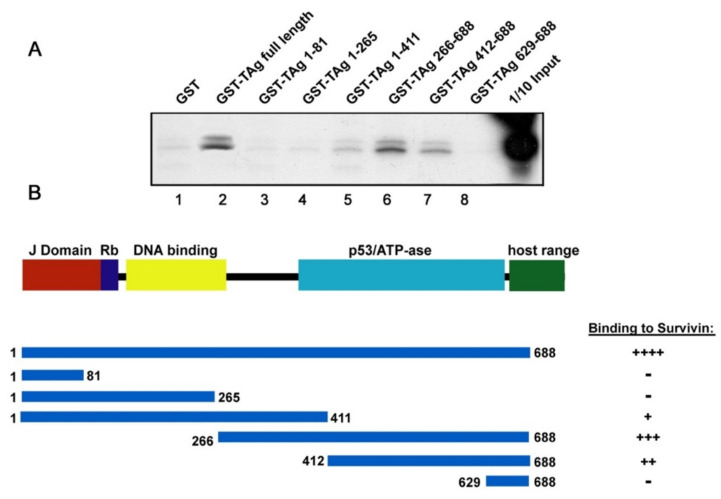
Survivin binds to the p53/ATP-ase domain of JCPyV T-Antigen. A GST-pulldown assay in which [^35^S]-radiolabeled in vitro-translated (IVT) Survivin was incubated either with GST, GST-T-Antigen full-length, or GST-T-Antigen deletion mutants bound to glutathione beads, was performed in order to determine the specific domain used by Survivin to bind to the early viral protein (**A**). Although binding exists with mutants 1–411 and 412–688, the strongest binding of Survivin appears to occur with the region spanning amino acids 266–688, which harbors the p53/ATP-ase motif of T-Antigen (A, Lane 6). A 1/10 input of [^35^S]-radiolabeled IVT-Survivin is shown. (**B**) Shows a schematic representation of JCPyV T-Antigen depicting some important binding motifs, with the spanning regions of the T-Ag deletion mutants depicted below. The ability of T-Antigen to interact with Survivin is shown to the right; +++ strong interaction, ++ moderate interaction, + weak interaction, - no interaction.

In order to determine the region where Survivin specifically binds to JCPyV T-Antigen, we performed a GST-pulldown assay using GST-T-Antigen full-length and a series of amino- and carboxy-terminal mutants of T-Ag fused to GST. We generated an [^35^S]-radiolabeled in vitro translated (IVT)-Survivin, which was later incubated with the respective GST-proteins. Survivin was detected in the fraction containing full-length T-Antigen (Figure 4A, Lane 2), but not GST (Lane 1), indicating that full-length T-Antigen specifically binds to Survivin. In contrast, the GST-T-Antigen carboxy-terminal mutants containing residues 1 to 265 lost its binding ability to Survivin (Lanes 3 and 4); however, mutants 1 to 411, 266 to 688 and 412 to 688 retained the ability to bind Survivin (Lanes 5, 6 and 7), suggesting that the active binding site for the anti-apoptotic protein is present in the same region of T-Antigen that contains the p53/ATPase motif (Figure 4B). Finally, the removal of residues 1 to 629 completely abolished the ability of T-Antigen to interact with Survivin (Lane 8).

## 4. Discussion

Mechanisms by which viral pathogens can overcome apoptotic cell death are a principal focus of recent virology research. Several studies point towards Survivin, a member of the inhibitors of apoptosis family of proteins, as one of the contributors to this process. Survivin up-regulation has been shown to be induced by viral proteins such as the E6 protein of human papillomavirus-16 (HPV-16) [41], Vpr from the human immunodeficiency virus-1 (HIV-1) [44], the latent membrane protein 2A (LMP-2A) from Epstein–Barr virus [40], the Tax protein from human T-cell lymphotropic virus-1 (HTLV-1) [45] and the Hepatitis B virus X protein (HBV-X) [42] with specific positive repercussions on viral proliferation. Within this context, we have previously demonstrated aberrant expression of Survivin in the infected oligodendrocytes and astrocytes of PML [24]. We have also detected increased levels of mRNA and Survivin protein by Northern and Western blot analyses in primary glial cell cultures upon JCPyV infection and, furthermore, JCPyV-infected cells were less prone to undergo physiological and/or experimentally-induced apoptosis when compared to uninfected populations [24]. All these data support the role of Survivin in JCPyV infection and in the pathogenesis of PML. Herein, we demonstrated the specific detection of Survivin by immunohistochemical analysis in the infected glial cells of PML but not in normal brain tissue, as well as the absence apoptosis and of caspase activity in the infected cells, supporting the concept of lytic, non-apoptotic death of infected oligodendrocytes.

Then, we tried to determine the mechanism for this Survivin up-regulation induced by JCPyV. Since Survivin levels are regulated through the cell cycle due to the activation/repression of its promoter, we studied the ability of the two non-structural regulatory proteins of JCPyV, T-Antigen and Agnoprotein, to transactivate the Survivin promoter. For this, we cloned two different lengths of the Survivin promoter sequence in a Luciferase reporter vector, and co-transfected primary astrocytes with each one of these constructs in addition to a JCPyV T-Antigen or a JCPyV Agnoprotein expression plasmid at different concentrations. Interestingly, T-Antigen, but not Agnoprotein, was capable of transactivating the Survivin promoter. Moreover, cells transfected with the longer length Survivin promoter were activated more efficiently than the shorter length construct, and this activation was dependent on the amount of T-Antigen present within the cells. Increased activation of the longer construct may indicate the presence of a T-Antigen-dependent activation site at the region, spanning between nucleotides −1066 and −667, and also that the region spanning the CDEs, CHR and Sp1 binding sites of the Survivin promoter is sufficient for T-Antigen-mediated transactivation. These functional data demonstrate, for the first time, that of the JCPyV proteins, T-Antigen alone has the potential to activate the Survivin promoter in vitro, and strongly point towards the viral regulatory protein as the cause of the Survivin up-regulation observed in PML. This is not surprising, since T-Antigen has been shown to activate many other cellular genes [46,47]. Moreover, up-and down-regulation of several genes (including cell cycle-related genes) can occur upon JCPyV infection in primary astrocytes [53], and one can hypothesize that some of these genes may become activated by T-Antigen. Nevertheless, the direct mechanism by which T-Antigen induces activation of the Survivin promoter remains elusive. Promoter activation could occur through direct binding of the viral protein to the Survivin promoter DNA or mediated through DNA-binding independent mechanisms, as has been proven for T-Antigen [46,47]. In this regard, it has been shown that wild-type p53 can induce transcriptional repression of Survivin through direct binding to specific promoter regions [54] and/or through chromatin modification of the promoter [55]. On the other hand, it is well-known that JCPyV T-Antigen can bind and sequester wild-type p53 [56]. Perhaps JCPyV T-Antigen may be able to induce Survivin activation through direct binding to p53 and thus causing the inhibition of the p53-induced repression of the anti-apoptotic protein. This mechanism of Survivin up-regulation by T-Antigen merits further study, including the analysis of both proteins at different cell cycle time points, at different stages of the viral lytic cycle of JCPyV, and in the presence and absence of p53. Mechanisms of regulation between these two proteins become more intriguing as T-Antigen is known to drive cells to enter G1/S phase, but, as mentioned earlier, it is in G1 where Survivin levels decrease the most.

An interesting finding about Survivin expression in PML is that it predominantly localizes to the nucleus of infected oligodendrocytes harboring inclusion bodies but remains mainly cytoplasmic in bizarrely shaped astrocytes (Figure 1). However, JCPyV-infected astrocytes also exhibit accumulation of Survivin in the nuclear compartment in vitro [24]. Moreover, here we have observed nuclear co-localization of T-Antigen and Survivin in JCPyV-infected primary astrocytes by double immunofluorescence. However, Survivin is considered a predominantly cytoplasmic protein that contains a nuclear export signal, with still uncertain functions within the nuclear compartment [50,51,52]. One interesting observation is that nuclear Survivin seems to enhance viral oncolysis, at least when used for adenoviral vector-specific therapies against tumoral cells [50]. Based on these observations, it can be suggested that DNA viruses, and specifically JCPyV, require nuclear Survivin for completing their lytic cycles. We hypothesize that Survivin may have a transient nuclear localization, depending on the stage of viral infection. It may be possible that, at the early stages of infection, where the highest levels of T-Antigen are available, Survivin could potentially bind more efficiently to T-Antigen and co-localize to the nucleus (as observed in vitro and in vivo in oligodendrocytes from PML samples in the current study), with a potential effect on viral replication, whereas at later stages of infection, when T-Antigen transcription decreases and capsid proteins are produced, Survivin may be in excess, and therefore retain/recover its normal cytoplasmic localization and function as seen in bizarre astrocytes of PML. Apart from the nuclear co-localization of both proteins, we have demonstrated a direct interaction of Survivin and T-Antigen by co-immunoprecipitation and the specific site for direct binding of Survivin and JCPyV T-Antigen to the p53/ATPase domain by GST-pulldown assay. That also could lead to the hypothesis that, after interacting with T-Antigen, Survivin is translocated to the nucleus, as mentioned above. Moreover, the Survivin/T-Antigen interaction may prevent Survivin ubiquitination and degradation, as occurs with the T-Antigen in complex with β-catenin [57].

Survivin up-regulation by viruses may not only have repercussions on viral proliferation but also on viral oncogenesis. Increased levels of Survivin have been constantly found in a wide number of malignancies, including viral-associated tumors. JCPyV is an oncogenic virus in animals and there is mounting evidence demonstrating its association with certain human neoplasms, especially brain tumors [8,58,59]. Some of the mechanisms of JCPyV-induced tumorigenesis are mainly related to the functions of early T-Antigen. As previously mentioned, T-Antigen can bind to p53 and alter its function, avoiding cells from preventing the accumulation of mutations, originally controlled by p53, and also leading to the de-repression of Survivin, which may be translated into cell resistance to apoptosis and progression into cancer.

Lytic infection of oligodendrocytes occurs in PML, whereas astrocytes acquire a transformed phenotype, becoming pleomorphic and atypical, reminiscent of glial malignant cells. Abnormal mitotic figures of astrocytes may also be observed [7]. The differences in the pattern of Survivin expression (nuclear in oligodendrocytes/cytoplasmic in astrocytes) suggest that Survivin may function differently in each cell type. T-Antigen-mediated up-regulation of Survivin may keep the cell alive and favor proper replication of the virus in oligodendrocytes, while interaction between T-Antigen and Survivin in infected astrocytes may alter Survivin function, promoting apoptosis resistance (with increased cell proliferation), abnormal mitotic spindle formation (with presence of abnormal mitoses) and, in conjunction with the transforming capacities of T-Antigen, promote the bizarre shape of cells as well as tumorigenesis.

## 5. Conclusions

In conclusion, we demonstrate, for the first time, the novel capability of JCPyV large T-Antigen to bind and activate the Survivin promoter at the level of transcription in primary astrocytes, in a dose-dependent manner, strongly supporting its role in the PML-associated Survivin up-regulation. Furthermore, we also found nuclear co-localization of T-Antigen and Survivin in JCPyV-infected cell cultures as well as direct interaction between these two proteins at specific T-Antigen domains. The reason for this interaction is still unknown but may have repercussions in the regulation of the viral cycle and in the replication of the JCPyV machinery within infected cells.

## Figures and Tables

**Table 1:** Clinical Data and immunohistochemical Results from PML Samples and Controls: Age, gender, diagnosis and associated diseases are listed. Results from immunohistochemistry quantification are shown for viral proteins VP-1 and T-Antigen, and for Survivin, and cleaved caspases 3 and 9, as well as results from the TUNEL assay. PML = Progressive Multifocal Leukoencephalopathy. AIDS = Acquired Immunodeficiency Syndrome.

No.	Age	Gender	Diagnosis	Underlying Disease			Immunohistochemistry			
					**VP-1**	**T-Antigen**	**Survivin**	**C/Caspase-3**	**C/Caspase 9**	**TUNEL Assay**
1	44 y/o	Male	AIDS-PML	HIV+/AIDS	**++++**	**+++**	**++++**	**-**	**-**	**-**
2	6 y/o	Male	AIDS-PML	HIV+/AIDS	**+++**	**+++**	**++++**	**-**	**-**	**-**
3	48 y/o	Male	AIDS-PML	HIV+/AIDS	**++++**	**++++**	**+++**	**-**	**-**	**-**
4	36 y/o	Male	AIDS-PML	HIV+/AIDS	**+++**	**+++**	**++++**	**-**	**-**	**-**
5	33 y/o	Male	AIDS-PML	AIDS + CNS Lymphoma	**++++**	**+++**	**++++**	**-**	**-**	**-**
6	73 y/o	Female	Non-AIDS PML	Renal Transplant	**++++**	**++++**	**++++**	**-**	**-**	**-**
7	46 y/o	Male	Non-AIDS PML	Lymphocytic Leukemia	**++++**	**+++**	**++++**	**-**	**-**	**-**
8	31 y/o	Female	AIDS-PML	HIV+/AIDS	**++++**	**++++**	**++++**	**-**	**-**	**-**
9	35 y/o	Male	AIDS-PML	HIV+/AIDS	**++++**	**++++**	**++++**	**-**	**-**	**-**
10	37 y/o	Male	AIDS-PML	HIV+/AIDS	**+++**	**++**	**+++**	**-**	**-**	**-**
11	48 y/o	Female	AIDS-PML	HIV+/AIDS	**++++**	**++++**	**+++**	**-**	**-**	**-**
12	44 y/o	Male	AIDS-PML	HIV+/AIDS	**++++**	**++++**	**++++**	**-**	**-**	**-**
13	57 y/o	Male	AIDS-PML	HIV+/AIDS	**+++**	**+++**	**++++**	**-**	**-**	**-**
14	35 y/o	Female	AIDS-PML	HIV+/AIDS	**++++**	**++++**	**++++**	**-**	**-**	**-**
15	45 y/o	Female	Non-AIDS PML	Chron’s Dis/Natalizumab	**++++**	**+++**	**++++**	**-**	**-**	**-**
16	52 y/o	Male	AIDS-PML	HIV+/AIDS	**++++**	**++**	**+++**	**-**	**-**	**-**
1	41 y/o	Male	HIV-Encephalitis	HIV+/AIDS	**-**	**-**	**-**	**+++**	**++**	**++++**
2	45 y/o	Male	HIV-Encephalitis	HIV+/AIDS	**-**	**-**	**-**	**+++**	**++**	**+++**
3	32 y/o	Male	Normal Brain	Drug Overdose	**-**	**-**	**-**	**-**	**-**	**-**
4	75 y/o	Female	Normal Brain	Breast Cancer	**-**	**-**	**-**	**-**	**-**	**-**
5	52 y/o	Female	Normal Brain	Myocardial Infarction	**-**	**-**	**-**	**-**	**-**	**-**
